# Supplementary material for: The Genetic Diversity and Dysfunctionality of Catalase Associated with a Worse Outcome in Crohn’s Disease
Source: Int J Mol Sci. 2022 Dec 14;23(24):15881. doi: 10.3390/ijms232415881 (PMC9785615; doi:10.3390/ijms232415881)
Supplement: Supplementary file 1 [file ijms-23-15881-s001.zip › ijms-1977688-supplementary.pdf]

<u>Supplemental Data 1:</u> <u>Sequences detailed of the CAT gene.</u>

Amplification Primers

**CAT DOWN**	ACGTTGGATGCTGCACAAAGGTGTGAATCG
**CAT UP**	ACGTTGGATGAGGCTCTTCTGGACAAGTAC

Extension Primers

**CAT EXT**	TGTGAATCGCATTCTTAGGCT

Amplification and Extension Primers of the internal controls

**ACTB-6 UP**	ACGTTGGATGTGAACCCCAAGGCCAACCG
**ACTB-6 DOWN**	ACGTTGGATGGGGTGTTGAAGGTCTCAAAC
**ACTB-6 EXT**	CTCAAACATGATCTGGGTC
**GAPDH-6 UP**	ACGTTGGATGCCCTGTTGCTGTAGCCAAAT
**GAPDH-6 DOWN**	ACGTTGGATGCAACGACCACTTTGTCAAGC
**GAPDH-6 EXT**	ttttTCAAGCTCATTTCCTGGTATG
**HMBS-7 UP**	ACGTTGGATGAGAGAAAGTTCCCGCATCTG
**HMBS-7 DOWN**	ACGTTGGATGAAGCCGGGTGTTGAGGTTTC
**HMBS-7 EXT**	gatGTTTCCCCGAATACTCC

Competitor Sequences

CAT comp

CTGCACAAAGGTGTGAATCGCATTCTTAGGCTaCTCAGCATTGTACTTGTCCAGAAGAGCCT

Competitor Sequences of the internal controls

ACTB-6 comp

GGGTGTTGAAGGTCTCAAACATGATCTGGGTCtTCTTCTCGCGGTTGGCCTTGGGGTTCA

GAPDH-6 comp

CCCTGTTGCTGTAGCCAAATTCGTTGaCATACCAGGAAATGAGCTTGACAAAGTGGTCGTTG

HMBS-7 comp

AAGCCGGGTGTTGAGGTTTCCCCGAATACTCCaGAACTCCAGATGCGGGAACTTTCTCT
